# Detection of Water Leakage in Drip Irrigation Systems Using Infrared Technique in Smart Agricultural Robots

**DOI:** 10.3390/s23229244

**Published:** 2023-11-17

**Authors:** Levent Türkler, Taner Akkan, Lütfiye Özlem Akkan

**Affiliations:** 1 The Graduate School of Natural and Applied Sciences, Dokuz Eylul University, İzmir 35390, Turkey; 2 İzmir Vocational School, Dokuz Eylul University, İzmir 35380, Turkey; taner.akkan@deu.edu.tr (T.A.); ozlem.karaca@deu.edu.tr (L.Ö.A.)

**Keywords:** robotics, water stress, irrigation, smart agriculture, IoT

## Abstract

In the future, the world is likely to face water and therefore food shortages due to reasons such as global warming, population growth, the melting of glaciers, the destruction of agricultural lands over time or their use for different purposes, and environmental pollution. Although technological developments are important for people to live a more comfortable and safer life, it is also possible to reduce and even repair the damage to nature and protect nature itself thanks to new technologies. There is a requirement to detect abnormal water usage in agriculture to avert water scarcity, and an electronic system can help achieve this objective. In this research, an experimental study was carried out to detect water leaks in the field in order to prevent water losses that can occur in agriculture, where water consumption is the highest. Therefore, in this study, low-cost embedded electronic hardware was developed to detect over-watering by means of normal and thermal camera sensors and to collect the required data, which can be installed on a mobile agricultural robot. For image processing and the diagnosis of abnormal conditions, the collected data were transferred to a personal computer server. Then, software was developed for both the low-cost embedded system and the personal computer to provide a faster detection and decision-making process. The physical and software system developed in this study was designed to provide a water leak detection process that has a minimum response time. For this purpose, mathematical and image processing algorithms were applied to obtain efficient water detection for the conversion of the thermal sensor data into an image, the image size enhancement using interpolation, the combination of normal and thermal images, and the calculation of the image area where water leakage occurs. The field experiments for this developed system were performed manually to observe the good functioning of the system.

## 1. Introduction

**Figure 1:**
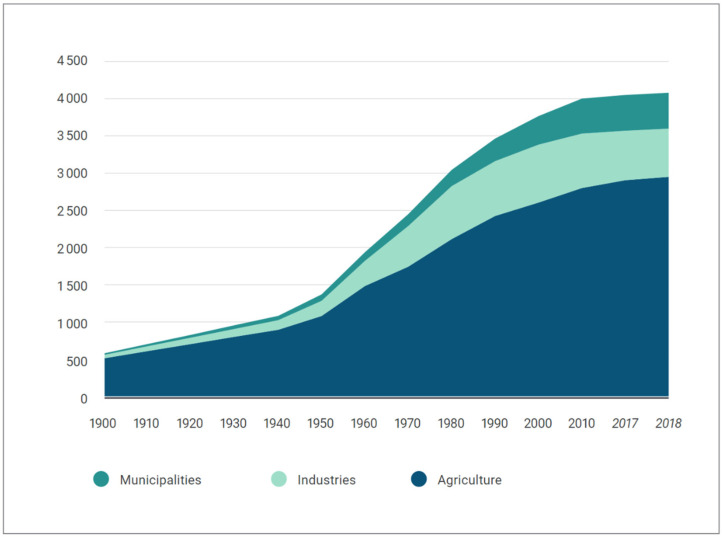
Evolution of global water withdrawals, 1900–2018 (km^3^/year) [3].

The demand for water supply and the consumption of water in the world is increasing, and access to water of good quality is becoming more difficult over time [1]. As a result of the decrease in the available water resources day by day and the excessive use of water, this demand is reaching a point that cannot be met [2,3]. Especially, as seen in Figure 1, a large amount of water is used in agricultural areas, and it becomes a necessity to use it in the most efficient way. Considering the future of the world and the inadequacy of people’s food resources, one of the solutions to minimize losses for the most efficient use of water resources is water saving [4,5]. Today, this event has become strategic for countries as well as for producers. Countries should take the necessary measures and make plans by making legal arrangements in this regard to ensure the continuity of their future and the needs of their citizens. It is not the right approach to look at it only in terms of food and water. For the world, which is expected to reach a population of 9.7 billion in 2050, the correct use of resources is a social and economic necessity [6]. In some regions and countries, the use of advanced technologies, especially in agriculture, has become a necessity, while in some regions, funds have been provided for studies on this subject from both national resources and foundations and unions. Over time, the development of the world has passed through various stages of industry. With the fourth industrial revolution, especially as a result of the integration of communication systems with control systems, a wide variety of application types in different application areas have appeared. With the use of Industry 4.0 applications and digitalization in the field of agriculture, Agriculture 4.0 has emerged [7]. Thanks to Agriculture 4.0 applications, it is now possible to create proactive and more efficient systems that can remotely monitor and/or control [8]. This is becoming a necessity; otherwise, insufficient water and food will cause disasters and chaos in the world. In order to prevent this situation, different technologies and many papers on this subject have been put forward [9]. While technological developments support many areas in agriculture, the control of the land with satellites [10,11,12], the control of data in cloud environments [13,14,15], the Internet of Things (IoT) [16,17,18,19,20], computer vision with the development of imaging systems, machine learning, and algorithms have started to be used intensively in agriculture [21,22,23]. In addition, information from all these studies have been collected and analyzed and studied using big data [24,25,26]. Thus, production is not considered only as instant harvest, and every piece of information received is used to make production more efficient. Today, this issue is not only considered for professional or large systems, but is also designed for lower-budget areas due to advancing software and electronic environments [27]. As can be seen, there are many studies and articles on this subject. The abundance of studies has also become a separate subject of study [28].

With the effective use of technology in agriculture, both the quality and quantity of products increase. It is not correct to limit the use of technology only to production. In order to increase quality and production, appropriate planting, fertilization, irrigation, and harvesting conditions should be provided [29,30,31,32]. These conditions can be controlled by human hands up to a point. However, the size of the land, the growing conditions of the product, climatic conditions, and the necessity of high-quality production will cause manpower to be insufficient at some point. Moreover, acting in a classical agriculture approach will cause the late detection of diseases, inadequate and inefficient use of equipment, and a high amount of manpower. Therefore, controlled agriculture is inevitable [33]. Fully controlled systems are seen in the field day by day. Even systems that control the irrigation system are being designed and implemented [34] to prevent errors. Small producers have started to use these systems to the extent that they are able. Especially in harvesting and irrigation, automation systems have reached small producers.

In this study, we focused on the design and development of electronic hardware equipped with a camera and thermal sensors to detect irrigation anomalies, which can be installed on a mobile robotic structure that scans the land area where irrigation faults occur, which are of great importance in agricultural production. It is very important for the quality and quantity of the product to irrigate the land at appropriate time schedules and to ensure the moisture content of the plant at the required times during the period from planting to harvest.

Irrigation does not only consist of releasing water to the land. It can be provided by giving water to the product or soil using different methods at different times. In this case, the technique and control of irrigation differs. During production, the humidity of the soil, plant, or air should be monitored according to the type of irrigation, and it should be checked whether the irrigation is proceeding well or not. Furthermore, although irrigation can be performed with automatic systems, it is also crucial to know which automatic irrigation system will be used. Irrigation control strategies should be determined, and control mechanisms should be put forward accordingly.

**Figure 2:**
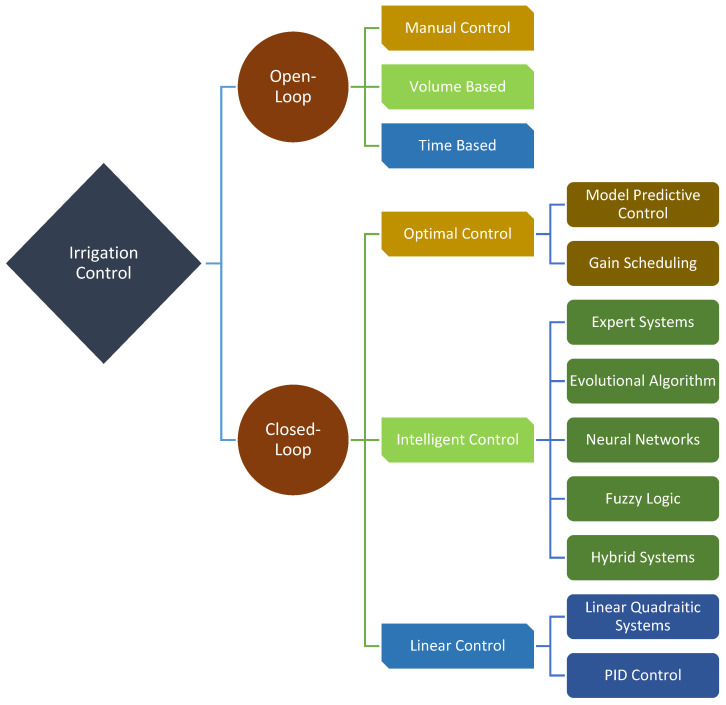
Classification of irrigation control strategies. Reprinted/adapted with permission from Ref. [35].

Bwambale et al. [35], who have conducted a good study on this subject, have defined control strategies in Figure 2. These control methods are mostly applied in the field. From the most basic method of the manual control of irrigation to the most advanced intelligent control, the purpose does not change. Providing water according to the product not only increases the quality but also provides savings [36]. Apart from this, it is also important which crop to choose, as well as where to plant it. In this way, we have the chance to make decisions about irrigation techniques [37,38]. It is not desirable to have an excessive or inadequate amount of water in the field than expected. The issue is the problems that can occur even when we do everything right. Considering that irrigation can be controlled in the field using any method shown in Figure 2, things do not always continue as expected. The larger the area, the more difficult it is to control every point of the control systems. Irrigation may not proceed as expected due to installation errors, malfunctions due to the dynamic operation of irrigation systems, weather conditions, damage caused by creatures that are not under control in the field, or damage to irrigation systems by conscious or unconscious people. In such cases, the water consumed at the irrigation point may be normal, but the irrigation point may shift, and the plant may be damaged. Therefore, it is necessary to scan water leaks in the field with mobile systems at certain time intervals. This study is not about predicting the water leakage problem beforehand; it is about creating an alarm so that the involved people can solve the problem in a short time to prevent water deficiency or excess. Even if problems can be detected by sampling methods, the reaction time after the occurrence of problems is expected to be minimum. Late reaction may cause irreversible losses in some cases. Increasing the number of sampling points in order to reduce losses will cause unnecessary costs [39].

**Figure 3:**
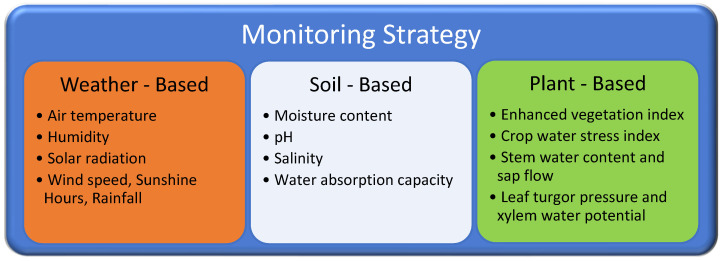
Monitoring methods in smart irrigation. Reprinted/adapted with permission from Ref. [35].

It would be optimistic to expect everything to proceed well. The establishment of security or control systems to ensure that the installed systems work without errors will eliminate such problems. At this stage, it is also necessary to understand how irrigation is performed. After the water is controlled at the desired point in the field, irrigation is monitored by different methods as shown in Figure 3 to understand whether everything is proceeding well in the agricultural area.

Considering that fixed systems cannot react to unexpected problems, additional systems are needed to recognize faults. Therefore, instead of fixed systems, mobile systems that control the terrain will be more efficient. The proposed electronic hardware and software for image processing in this study, such as for water leakage detection, was designed considering its integration with such a future mobile robotic structure. With this approach, it is possible to identify the common problem areas, including water ponding, and mark them for manual or automatic resolution while moving around the land area.

**Figure 4:**
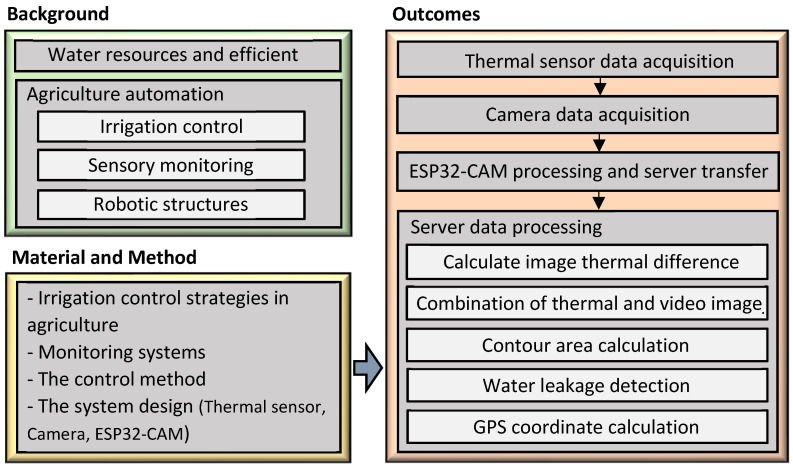
The layout of the study.

In the introduction, we mentioned an effective agricultural irrigation system and method for minimizing the water leakage, especially to be used in smart agriculture robotics. In the next section, general information will be given on agricultural irrigation control strategies, monitoring systems, control methods, and the proposed system design. The system design covers thermal sensors, a camera, and an ESP32-CAM Wi-Fi Bluetooth Development Board. In section three, the results of the study will be given. The results include image data transfer from the camera and thermal sensor, data processing by the server, and water area detection. In the final section, the conclusions and discussion will be given. The layout of the study is given in Figure 4.

## 2. Materials and Methods

### 2.1. Agricultural Irrigation Control Strategies

Today, the decrease in freshwater resources has made it obligatory to use water sparingly. To this aim, irrigation systems suitable for the type of product, climatic conditions, size and conditions of the land, and geographical conditions of the land are used. Irrigation in agricultural production is performed by natural means or by human hands. Natural irrigation relies on timing with respect to weather conditions, often leaving it to chance. Although this method is a very primitive method and has a direct effect on the quantity and quality of the product, insufficient or excessive irrigation can cause damage that will leave the producer in a difficult situation. However, the producer can be organized to minimize these undesirable situations using monitoring systems.

**Figure 5:**
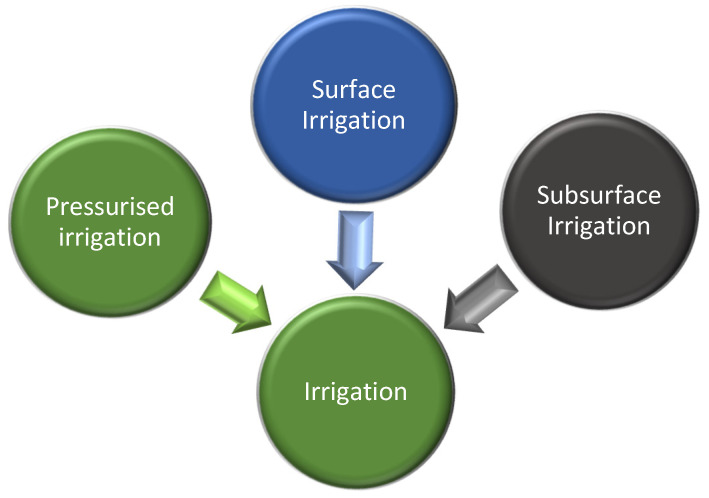
Irrigation methods.

Although excessive irrigation can damage the plant, there is basically no unknown situation in the field. In short, since irrigation is not adequately controlled, it is unnecessary to prevent it or to check whether there is excess water by travelling to various points of the land. In man-made systems, irrigation is carried out by considering the plant’s need for water. Here, water can be provided directly to the plant, or it can be provided in very close proximity to the plant in a way that it can reach the roots of the plant. The application depends a little bit on the possibilities available on the land. Accordingly, agricultural irrigation is realized using three different methods. While some of these methods are controlled by modern systems, some of them are not controlled much due to the advantages of land conditions. It is possible to see these in Figure 5 [40,41].

Surface irrigation systems are systems where irrigation is performed by utilizing the conditions of the land. Here, water moves along a natural slope and flows into canals. Water is provided to the plant by ponding on the land. This method, called “basin” or “flood” irrigation, which is widely used in classical agriculture, is a method that can cause the excessive expenditure of water and evaporation. In this method, water saving is not often pursued [42]. Water is released to the land, and it is assumed that the soil receives the water it can take. Excess water is either lost by evaporation or it finds its way into the soil and disappears before reaching the plant. According to the studies on farmer preference, the producer mostly prefers this method because of the product need, because it is easy, or out of necessity [43]. In subsurface irrigation, water is provided to the roots by the effect of capillary forces (capillarity) under the soil. It has been determined that this method provides significant benefits to plant development, and while there is a great decrease in the amount of water used, it causes extra investments, and different problems may occur due to unused elements in the water [44,45]. Another method is pressurized irrigation. Here, water is delivered to the plant by increasing the pressure to a certain value. The pressure is created by mechanical systems or by delivering water to the plants from a point potentially higher than the irrigation point. Water is usually delivered to the plants either as drip irrigation or with the help of springs according to the type of product. In this system, water is used more efficiently than in the basin irrigation system. When considering irrigation efficiency, it is not advisable to accumulate excessive water at a single point or to interrupt the planned watering process, which ensures that the plants receive an adequate amount of water during irrigation. Doing so may lead to plants being left without water. As in Figure 3, we can try to avoid this problem by controlling irrigation using the methods described by Bwambale et al. [35].

Although smart systems constantly control the land and try to provide water according to the thirsty plant or soil, it does not matter how much water you provide when there is a problem with the irrigation, because the necessary amount of water will not reach the desired point. In such systems, the necessary sensors can partially detect this problem.

Our goal here, obviously, is pressurized systems that can be controlled. The basin irrigation system is not overly concerned with water conservation. In subsurface irrigation systems, it may not always be possible to detect water leaks. Using the method in the system we designed, we tried to detect leaks in drip irrigation systems. Water was found naturally outside the plant, as irrigation was delivered to every point of the system by sprinklers. Therefore, problem detection in irrigation with sprinklers could not be realized with the designed system.

### 2.2. Monitoring Systems

Considering the monitoring systems depicted in Figure 3, two potential scenarios arise within the system being examined: irrigation is either excessive or insufficient. The excess of irrigation is manifested by its appearance in the soil in an area different from the plant, while the lack of irrigation is manifested by the poor development of the plant.

Irrigation in modern systems is carried out in open and closed loops. In open loops, irrigation is provided to the system on a time or volume basis. Whether the irrigation is proceeding correctly in the field is either determined by the employees or ensured by the sensors in the field. Whether adequate irrigation is carried out in these systems, whether on a time or volume basis, is mostly based on the crop, using the sampling method, and utilizing experience. In closed loops, fuzzy logic [46,47], PID irrigation control [48], neural networks [49,50,51], linear quadratic systems [52], evolutionary algorithms [53], and hybrid systems are used to control irrigation and ensure the most efficient irrigation. In these systems, irrigation is performed according to the condition of the plant, soil, and climate. In addition, drainage water is utilized in irrigation when required, and the accuracy of irrigation is controlled. In this type of system, there are natural mechanisms to prevent unnecessary water loss. However, these mechanisms will be of secondary importance as watering will take priority when the plant signals insufficient water. In some cases, water loss may not be detected by the algorithms or may be detected too late.

**Figure 6:**
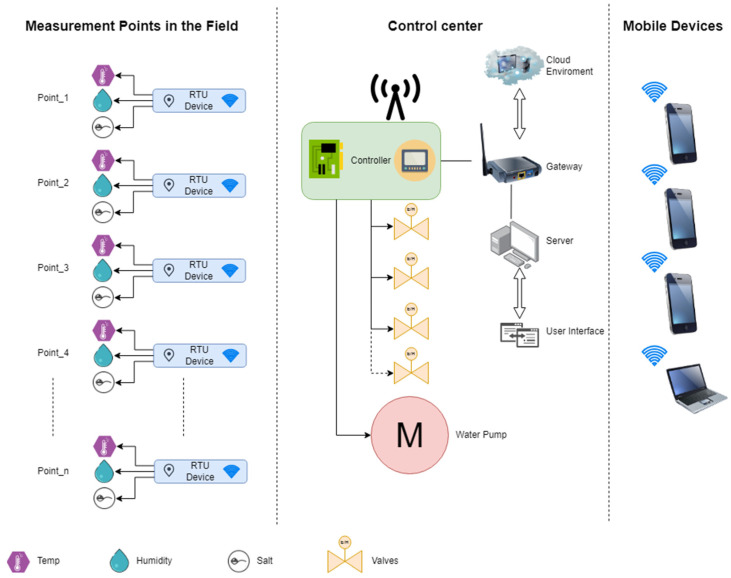
An example supervisory data acquisition and control system.

For these reasons, the control of irrigation, whether by an open- or closed-loop system, should be conducted using not only algorithms but also control mechanisms implemented at the farthest endpoints. This means sensors at many points in the field. The sensors to be used can continuously control the soil moisture, pH balance, and salt ratio, and can inform the system and detect errors. These devices are frequently used today. In the example system in Figure 6, a central control system and remote end units evaluate the data coming from RTUs, such as soil salt, pH, and humidity, and operate their valves and motors according to the parameters determined by the server or the panel on the controller. In addition, controller parameters and RTU (remote terminal unit) values are stored in the cloud environment, which can be accessed by mobile devices and when necessary, authorized persons can intervene from outside the field.

Naturally, in this system, there is a need for enough sensors to operate and control the entire system without error. The fact that all RTUs can talk to the CPU eliminates the possibility of being wired due to changes in production periods and products in practice. Therefore, the system should be built on wireless networks. Moreover, additional devices will also be required to act as repeaters if the distance between the sensors or the control center is exceeded. Even with all these systems installed and working perfectly, there is no guarantee that there will be no problems, and if there are, additional mechanisms will be needed to pinpoint the exact location of the problem. It may be possible to avoid the problems associated with traditional systems by adding mobile robot technology. Mobile robots travelling around the field and detecting the point where the leakage is located is the most suitable solution for the system to work properly. This avoids situations that the controller software does not recognize as errors.

### 2.3. The Control Method

**Figure 7:**
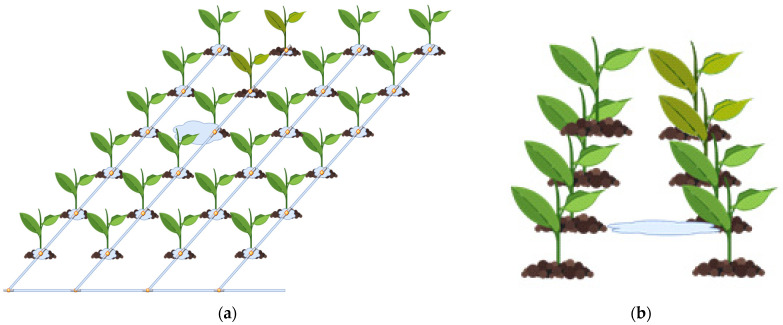
Example water leakage. (**a**) Plant furrow view. (**b**) Detailed plant view.

After identifying the problem and the solution steps, it is necessary to determine how to solve it. The main question is how the control system investigates the land and detects the problem. It should be noted here that this process must be performed both quickly and accurately. After all, wrong decisions cost time and money when looking for a fault that needs intervention. In this sense, it should be considered what happens when there is an error in irrigation. If there is a blockage at the irrigation point, then water cannot reach the plant and causes anomalies in plant growth. These abnormalities, often called stress, are usually caused by heat, but mineral deficiencies, cold and frost effects, and other factors have also been found to cause them [54]. The most important of the other factors is the lack of water. Moreover, water excess also causes stress. In Figure 7, an example water leakage problem can be seen. In the view of plant furrows, the leakage area causes excess water, which is a problem for the nearest plant, and triggers a lack of water for the subsequent plants in the line of the water furrow. In the detailed view of the problematic area, the plants experiencing water deficiency can be seen.

If the water does not reach the desired points, then it can be determined that the calculated water has not reached the plants by reviewing the algorithms that determine the water and drainage water provided in closed systems. In addition, the deformation of the irrigation canal at a point means that the water will not move from this point and will accumulate and pond. In this case, the plants here remain in contact with excess water and become stressed again. In the continuation of the water channel, plants that cannot be contacted by water are stressed due to drought. Again, advanced systems can recognize this problem from drainage or by seeing an unexpected increase in water. Although it can be determined by monitoring systems whether water goes into the land more or less than desired, where the problem is or when it will be noticed are important considerations. Mobile systems come into play in this case. They can determine whether everything is proceeding well by travelling around the field when the problem occurs or before the problem occurs. Moreover, when they find the problem, they can intervene as soon as possible by informing the central system.

It is important to define what the mobile system looks for in the field and how it searches. In short, to know how the mobile system detects the problem in the field is required. If we consider that the water supply to the plant in the land is cut off when there is a problem, then finding the cut-off point means finding the problem. For this, it is necessary to find the point where the water accumulated. If the designed mobile system detects the area where water accumulates in the field or where there is more moisture than expected, then it will naturally detect the source of the problem. For this, detecting excess moisture in the soil solves the problem in the fastest and most accurate way. However, this raises the question of how quickly moisture detection using moisture meters will solve the problem. Considering the size of the field, the question of how many points the moving vehicle will have to examine if it stops at one point and starts moisture measurement is also important because it will cause a loss of time.

In this study, the problems encountered in drip irrigation, which is one of the most widely used systems in agricultural irrigation, is discussed. Although the technique to be used can support the capillary method with some subsurface water, trials have not been carried out in this kind of irrigation system. The reason for choosing this irrigation system is that it is the most widely used system, but also the one with the possibility of problems. Since the detection of irrigation leaks will be slow due to the need for long-term measurements using humidity sensors, faster humidity detection using a thermal camera while moving represents the difference in this study. Water excess can easily be found and confirmed using image processing thanks to the powerful microcontrollers of today. In this regard, another approach by Farkas was the method of irrigation control using the stress effects on the plant and computer vision [55]. However, what we do here will identify the soil where there is a water leakage, and the plant stress method is not required to achieve our goal.

### 2.4. The System Design

A camera, thermal sensor, and microprocessor were used in the mobile electronic system design. The data from the camera and the thermal sensor were transferred to the server via the microprocessor and processed there. Here, the camera and thermal sensor received the same image from the area investigated, which eased the decision making regarding the detection of water excess. Below, the system components will be explained separately.

#### 2.4.1. Thermal Sensor

In this study, AMG 8833, which is an 8 × 8 array thermal sensor that can act as a camera, was used to track thermal traces for a purpose-built structure. The thermal sensor specifications are in Table 1. This temperature sensor sends a temperature value that can create a representative image of 64 pixels. The readings are easily acquired using the I^2^C serial protocol. Although it is referred to as a camera, the resulting data are sensor values in the temperature range of 0 °C to 80 °C (+32 °F to +176 °F). The result of the software is that the 64 generated datapoints are converted into an 8 × 8-pixel image.

The biggest reason for using this sensor is that it fully meets the temperature to be utilized in the field. In addition, the communication protocol used is the well-known I^2^C, which is generally used for effective serial communication between sensors and microcontrollers. The fact that many microcontrollers support this protocol has been effective in the selection of the sensor. In addition, its stable operation and good price/performance ratio also played a positive role in the selection. However, the low matrix value of the sensor, such as 8 × 8, is its disadvantage.

Although it is possible to use this sensor directly, it can easily be interfaced with microprocessors using breakout boards. The breakout board simplifies system integration by providing soldering, serial addressing, and assembly. Although there are many software libraries that can be used with this sensor, the code has been written using the sensor datasheet instructions for better software integration and faster execution. In addition, the reading angle of the sensor being 60° is another issue to be considered for selecting the required view distance.

#### 2.4.2. Camera

The OV2640 color image sensor is used in the design as a camera. This camera has a 2 MPixel resolution with a total image array of 1632 × 1232 pixels. However, a lower resolution of 320 × 240 pixels was used in the design to ensure processing speed. The main features used in the OV2640 image sensor are 10-bit A/D converters and a digital signal processor (DSP), with a UXGA/XGA resolution of 15 fps, SVGA 30 fps, and CIF 60 fps allowing images to be taken. It also allows for automatic image control functions including automatic exposure control (AEC), automatic gain control (AGC), automatic white balance (AWB), automatic band filter (ABF), and automatic black-level calibration (ABLC) of the image. The main reason for using this camera sensor in this study is that it comes with this breakout hardware and is compatible with the ESP32 processor. In this way, the connections of the camera can easily be made using the socket on the breakout [57].

#### 2.4.3. ESP32-CAM Wi-Fi Bluetooth Development Board

In this study, the images were not processed on the microprocessor but transferred to the server and analyzed there. To ensure real-time data processing on the server, a high data transfer rate and appropriate communication distance were planned. Furthermore, given the system’s mobility requirements, it was designed to achieve low weight and high energy efficiency. These design choices were made to facilitate the transfer of the system to lightweight robots, such as drones, in the next stage of the study. Although there are numerous microprocessors or controllers that could be used for this purpose, the ESP32-CAM [58] was chosen as it contains all the necessary components. The presence of the Wi-Fi module, the camera module, and SD card socket for data recording meet the requirements of this study. Additionally, the availability of multiple data communication protocols is valuable for adding new sensors and creating new wireless interfaces in the future. The board can be programmed in languages such as C and supports a variety of other languages such as MicroPython [59].

## 3. Results

**Figure 8:**
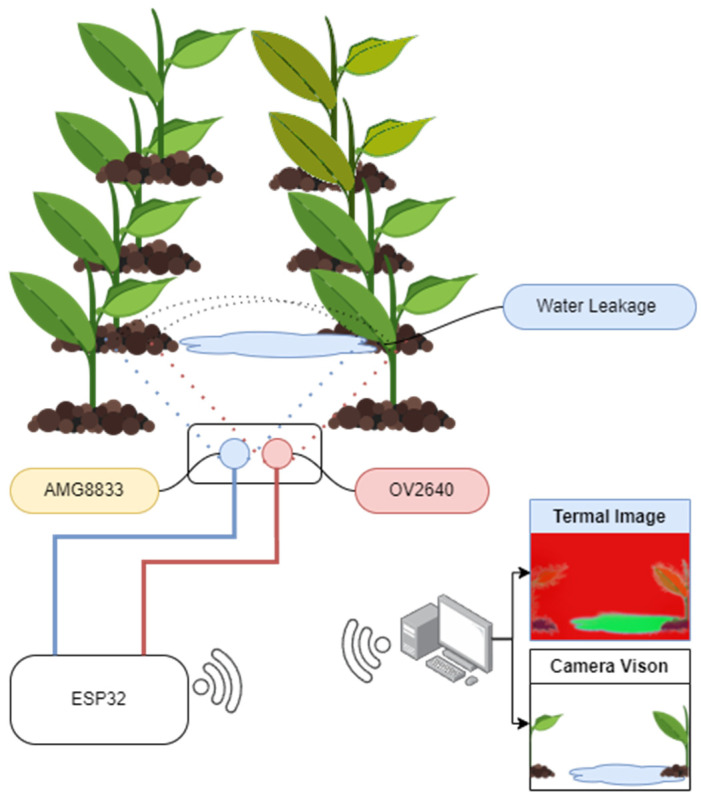
Schematic representation of the system.

In the designed system (Figure 8), the data from the camera and thermal sensor were collected in the ESP32-CAM and transferred to the server computer via Wi-Fi and processed there. The developed server software was able to detect water leakage that occurred during irrigation by analyzing camera data. Thus, the use of thermal cameras instead of traditional humidity sensors was successfully implemented in detecting water leaks. The transfer of the images to the server, processing on the server, and the results of the experimental study will be presented below.

### 3.1. Transferring Images Using ESP32-CAM

There are two different pieces of information received by the mobile system. The first is the data from the camera and the other is the data from the thermal sensor. The data from the camera have direct video images. These images can be viewed directly using its software library without any processing. The thermal sensor data consist of float-type values loaded in two bytes with a resolution of twelve bits, as shown in Table 2. The first eleven bits of these data denote the data values, whereas the twelfth bit specifies whether the number is positive or negative. The thermal sensor data with an 8 × 8 array, 128 datapoints between addresses 0x80–0xFF in total, are combined as 64 float type numeric data in list type. The video images were transmitted to the server via web server port 81 and the thermal sensor data were transmitted via web socket port 82 through two different channels over the wireless network.

**Figure 9:**
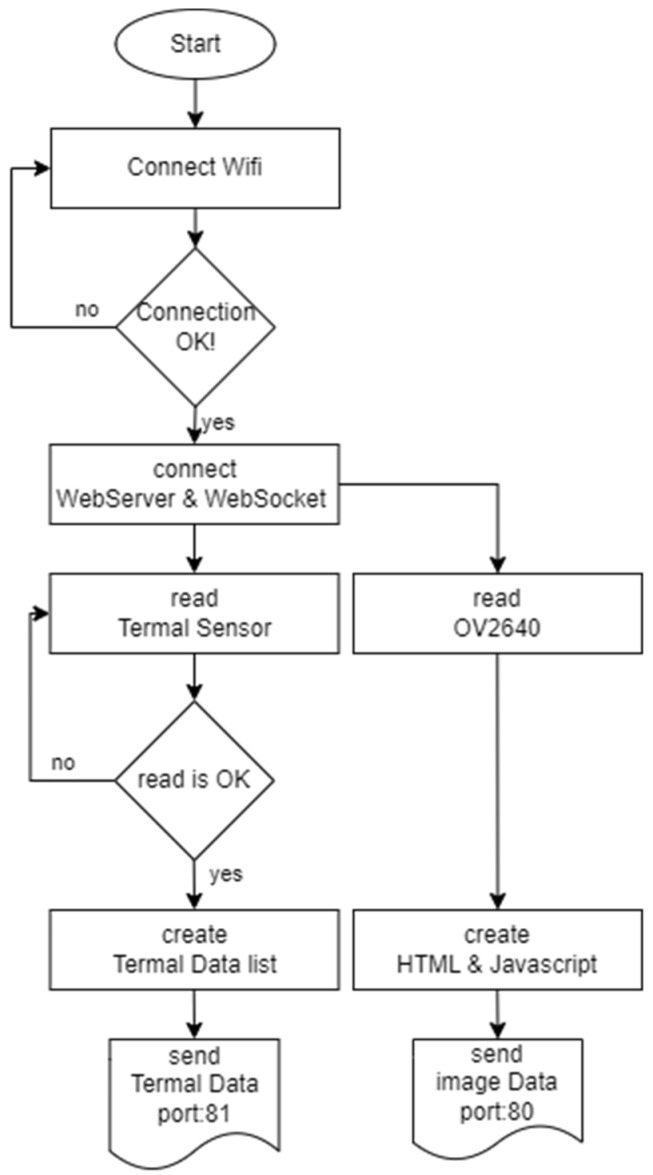
ESP32-CAM program algorithm.

Video images and thermal data can also be sent from the same port, but the two-channel transfer method was used because distinguishing these two types of data and analyzing them on different devices was necessary. The video image from the camera was sent in 320 × 240-pixel resolution. This information and all the setting parameters of the OV2640 can be adjusted by changing the parameters on the server PC if required. The thermal sensor data comprised a total of 256 bytes of data, but the first 128 bytes contained data not needed for the image operation, such as interrupts, thermistor output values, etc. The remaining 128 bytes contained the necessary thermal image data, and after the data acquisition of these 128 bytes by ESP32-CAM, the server transferring was initiated. These operations were implemented using the algorithm shown in Figure 9.

### 3.2. Data Processing on the Server

The data transferred to the server from two different ports were processed separately here, while the data were combined and visualized to ensure consistency. The initial 8 × 8-pixel image represented 64 numerical variables consisting of temperature values. A color scale with 64 temperature levels was established to visualize the image. The first color in the scale represented the lowest temperature, while the last color represented the highest temperature. Although the thermal sensor can measure in the range of 0–80 °C, it is not necessary to use the entire range for the temperature values to be measured in this study. The land is not expected to be as low as 0 °C during the sowing and harvesting period, and a maximum of 80 °C is not possible in the sown area even on the hottest days. Therefore, in this study, the temperature range was determined as 25–45 °C. As mentioned above, the lowest temperature color showed 25 °C, while the highest temperature color showed 45 °C. In this way, the 64 incoming datapoints were converted into 64 color values according to the temperature value. After that, an 8 × 8-pixel image was formed consisting of colors by arranging the 64 datapoints in an 8 × 8 array format.

However, this size of image is insufficient for image processing and is too small to interpret. Therefore, the image was enlarged to a resolution of 320 × 240 pixels using the OpenCV [60] interpolation method successfully. Here, the bicubic interpolation [61] method was preferred for expanding the image area by fitting the proper value for newly added pixels. In this method, during the resampling of the image, it is reconstructed by selecting the nearest neighboring pixel values to the gaps. Although this method is a little slower in terms of computational speed when compared to the other methods, this difference is not noticeable for the purpose of this study. The mathematical model used for interpolation is shown in Equation (1) [62]:

(1) $$p\left(x,y\right)=\sum_{i=0}^{3}\sum_{j=0}^{3}a_{ij}x^{i}y^{j}$$

It is incorrect to assume that every irrigation point is a leaking point. To avoid such a situation, it is also important to consider the location and size of the leakage point. In the case where an 8 × 8-pixel image is used, due to the low resolution, not every anomaly observed will potentially indicate a water leak. With the interpolation method applied, the 8 × 8-pixel image becomes sufficiently clear and visually useful. Thus, when the image resolution is increased, it is appropriate to evaluate whether the irrigated area has received more water than necessary by calculating the area from the measurement points. For this, the calculation of the area where the water spreads using the contour’s calculation in OpenCV can be made from the image. If this area is more than the desired size, then it is evaluated as leakage; otherwise, it is considered normal irrigation.

### 3.3. Experimental Study

**Figure 10:**
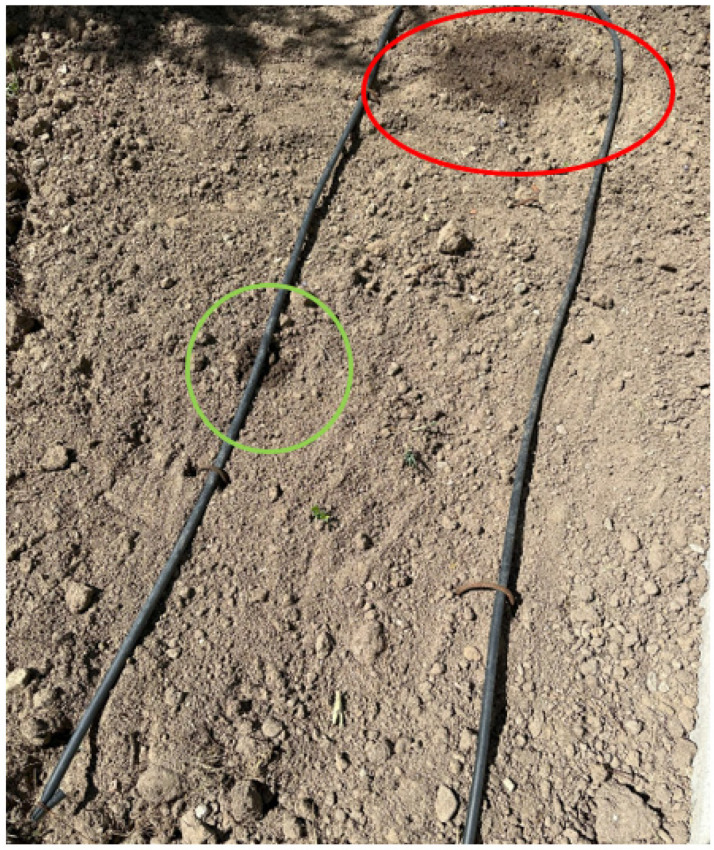
Test area (**red circle**: water leakage point, **green circle**: normal watering point).

For the experimental study, an area irrigated with the basic drip irrigation technique was selected in the field. The condition of the soil was dry, and the weather condition was hot and not very humid at the time of the test. As shown in Figure 10, a sample normal irrigation hole and a sample defective irrigation hole were created on the irrigation pipe in the test area. A properly functioning irrigation hole provides sufficient water to the plant, and this irrigation pattern is indicated by the green-colored marking. Abnormal watering, on the other hand, releases more water than is needed, resulting in water leakage, which is indicated by the red-colored marking.

**Figure 11:**
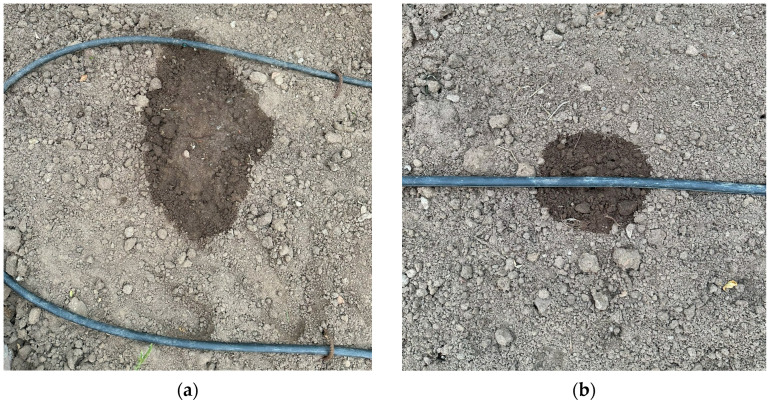
Test points. (**a**) Water leakage point. (**b**) Normal watering point.

The video image stream of the points in the selected test area was taken with the system prepared as shown in Figure 11. In addition, real-time thermal images were also captured via web socket at these points.

**Figure 12:**
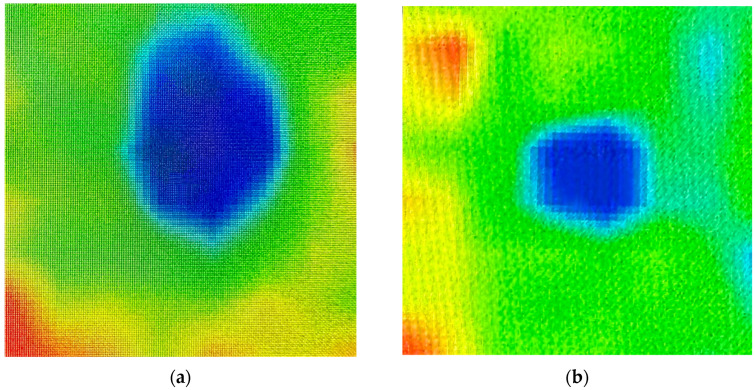
Test points thermal image (The colors represent heat level, blue: cold, red: hot). (**a**) Water leakage point. (**b**) Normal watering point.

The Figure 12 shows thermal images of leakage and normal watering conditions at test points.

Naturally, each image taken must have the same distance to the test point. This is due to the need to ensure the consistency of the results of the measurements. Otherwise, i.e., if each measurement area is selected with a different height, then the leakage point may not be detected, or normal irrigation points may be misinterpreted as leakage points. Since the distance between the furrow was 0.65 m and the camera’s angle of view was 60°, the distance (height) from the soil level was calculated as approximately 0.6 m from the division of half of 0.65 m to the tangent of half of 60°.

**Figure 13:**
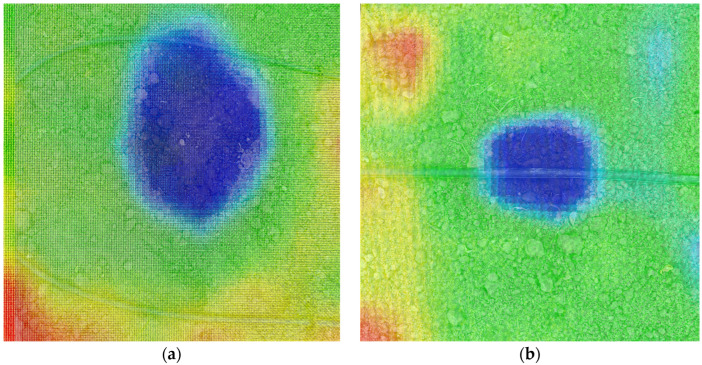
Combination of thermal image and video image (The colors represent heat level, blue: cold, red: hot). (**a**) Water leakage point. (**b**) Normal irrigation point.

In this study, since there were no obstacles in the field and there were no plants on it, it was possible to take the measurements manually from a pre-calculated height of 0.6 m. In this measurement, by superimposing thermal data and normal camera images captured from the video feed of the OV2640 camera sensor in the software, faulty irrigation points in the soil were more clearly revealed (Figure 13).

**Figure 14:**
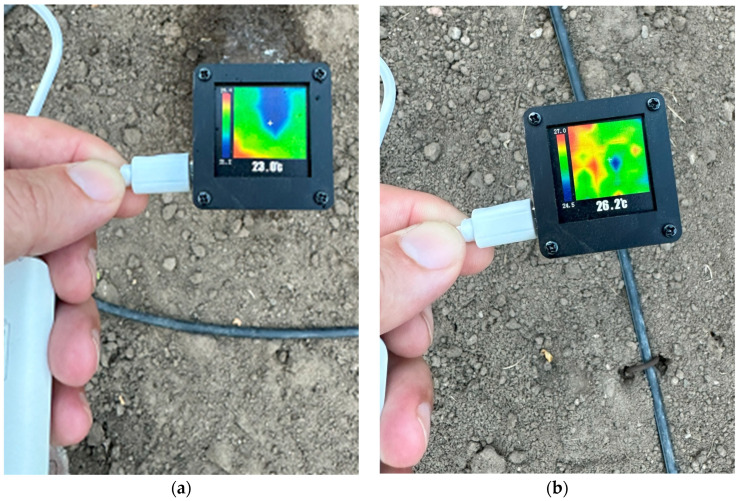
Control with another thermal sensor. (**a**) Water leakage point. (**b**) Normal irrigation point.

The operation of the designed system was verified and tested using a reference thermal sensor module currently available on the market (Figure 14). A temperature difference was evident between the irrigated and dry areas as shown in the figure.

**Figure 15:**
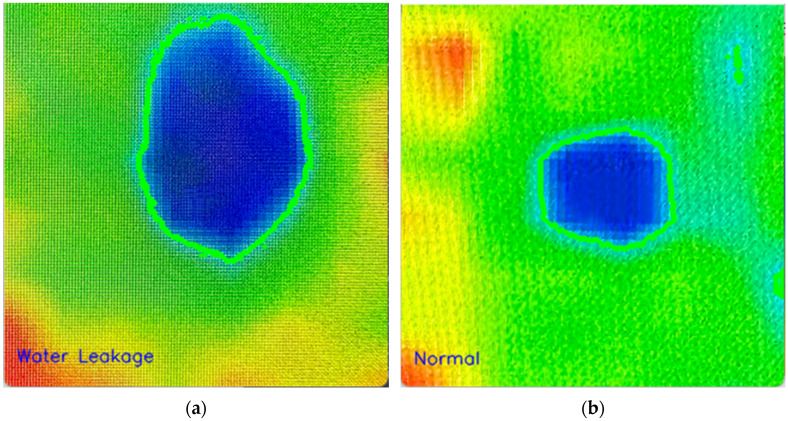
Field control of test irrigation points (The colors represent heat level, blue: cold, red: hot). (**a**) Water leakage point. (**b**) Normal irrigation point.

What needs to happen after obtaining these images is to determine which image represents leakage and which image represents normal irrigation. As can be seen in Figure 15, this is the process of calculating the area from thermal images with the contourArea command in OpenCV. Here, which is labelled as water leakage and which is normal irrigation can be determined according to a parameter to be determined.

When the water leakage area is detected, the point where the leakage is located can be marked by the mobile system, GPS coordinates can be recorded, or the user can be informed via a generated alarm message.

## 4. Discussion

The importance of this study is to obtain better irrigation in agricultural areas with the help of detecting the leakage of water. The leak is detected using the thermal anomalies occurring in the irrigation area. It has been proven that this is possible using a camera and thermal sensor together with the proper arrangements in the agricultural area. It is indisputable that this method is both cheap and fast compared to other humidity control methods. In addition, the sensors required for the operation of the system are cost-effective and easy to use in this study. Leakage control with thermal cameras produce a faster and more flexible solution than temperature and humidity sensors. The conventional water sensors that detect one-dimensional signals require a long measurement time in the order of minutes for a consistent measurement. This may not be a suitable solution as it extends the process of scanning agricultural land for water. Monitoring a single physical variable would also not provide flexibility in terms of multi-tasking compared to a camera. In addition, with the use of cameras, not only water leakage detection but also other image-recognition-based studies can be carried out in the field.

Larger areas can be scanned with more advanced electronic systems. In this way, control can be carried out even faster. If these systems are combined with autonomous multi-robots, then control in larger fields can be provided with great ease. It is possible to design this autonomous system to be used with a wheeled land vehicle moving on the ground as well as with the help of drones moving in the air.

While designing the system, the land selected was determined as areas where there was no vegetation or very small vegetation. In addition, the reaction of the system to dense plantings or large plants has not been studied. In dense vegetation cases, both detecting water leakage using thermal imaging and the deployment of ground or aerial mobile robots carrying the thermal imager will be challenges to address for future works.

The measurements here were acquired with a simple moving mechanism to keep the height of the camera and thermal image constant at a height of 0.6 m and to keep the viewpoint unchanged. The aim of this study was to test the functionality of the designed and realized electronic diagnostic system. The fully controlled and autonomous structure of the system will be addressed in the next iteration.

It is foreseen that there may be some problems with leakage control in thermal system. For example, it is thought that there may be erroneous readings due to the structure of the vegetation and that the shadows of large plants may cause errors in the readings. It is also considered that the thermal sensor may miss temperature anomalies in the soil due to the surrounding ambient temperatures in very hot weather during operation, although not very often.

## 5. Conclusions and Future Works

The detection of water leakage was studied, and successful results were obtained on the use of water resources efficiently for agricultural irrigation purposes. A low-cost computerized embedded system was designed to collect image and thermal sensor data to evaluate the soil wetness conditions. Here ESP32-CAM was the main embedded computing module combined with an OV2640 camera module. The thermal sensor selected was the easy-to-use and low-cost AMG8833 8 × 8 array thermal sensor. The sensor data were transferred to a personal computer using wireless data transfer to evaluate the water condition. With the help of the normal camera sensor and proper interpolation method, the resulting thermal image was obtained successfully on the PC side. The evaluation and decision phases were realized on the resulting image using the greater computer processing power of cloud computing. Thus, the detection of water leakage was achieved.

The main aim was to observe the system’s functionality and prove that the combined normal and thermal images worked for water leakage detection. Here, the challenges with algorithms in the aspect of software development were well overcame. The system’s statistical performance, which is not quite possible to test manually, can be evaluated when the system is placed on an automatic scanning mobile robot. Future work will cover the intensive tests of the developed system of this study.

As a future work, in addition to the detection of leaks from thermal images in the leak detection study, the data from the camera can be processed by new machine-learning software running on the server. Thus, this new software can be used to confirm that the temperature change in the soil is due to water leakage. For this, a dataset will be prepared to train test images and a model will need to be created. With this model, more precise decisions can be made by examining the objects, not only depending on the data from the thermal sensor. This helps the thermal system with both dense plants and complex terrain samples. In addition, in this way, the thermal system prevents the object recognition system from making the wrong decision and helps make the right decision. Apart from this, the thermal system eliminates the misinterpretation of the image coming to the camera for the dataset as the effect of shadow or light during the movement of the mobile system in the field.

Another future work will be about automated data acquisition using a ground agriculture robot and potentially a drone robot to obtain automated scans of the agricultural land. This automatic robotic system will also allow the sensor data acquisition process to be more reliable when compared with the manual approach. It is also possible to obtain the current navigation position if required, in addition to the identification of the problem. If this system is placed on a robot that scans a given area, then the location of a water problem could be obtained automatically by considering any detected abnormal situation. Moreover, this robot can also carry a more powerful computer such as Raspberry pi or industrial mini personal computers inside. Thus, the data are collected and processed in the same robotic system as in edge computing.

## Figures and Tables

**Table 1:** AMG8833 Infrared Array Sensor specifications, Grid EYE 8 × 8 Preference [56].

Item	Performance
Number of pixels	64 (Vertical 8 × Horizontal 8 Matrix)
External interface	I^2^C
Frame rate	Typical 10 frames/s or 1 frame/s
Output mode	Temperature output
Temperature range of measuring object	Low Gain: 0 °C to 80 °C; +32 °F to +176 °F High Gain: −20 °C to 100 °C; −4 °F to +212 °F
Applied voltage	3.3 V
Viewing angle	Typical 60

**Table 2:** Temperature information of a pixel coming from the thermal sensor [40].

Address	Register	R/W	bit7	bit6	bit5	bit4	bit3	bit2	bit1	bit0	Initial Value
0x80	T01L	R	T7	T6	T5	T4	T3	T2	T1	T0	0x00
0x81	T01H	R	--	--	--	--	+/-	T10	T9	T8	0x00

## Data Availability

Data were generated by the designed system using the sensors, there is no need to share it. In other researches, it is already required to be produced specifically for the study itself.
